# Quantitative Assessment of Birefringent Skin Structures in Scattered Light Confocal Imaging Using Radially Polarized Light

**DOI:** 10.3390/s130912527

**Published:** 2013-09-17

**Authors:** Babu Varghese, Rieko Verhagen, Altaf Hussain, Clemence Boudot, Qiangqiang Tai, Siqi Ding, Jasmin Alexandra Holz, Natallia Eduarda Uzunbajakava

**Affiliations:** Care & Health Applications Group, Philips Research Europe, High Tech Campus 34, 5656AE Eindhoven, The Netherlands; E-Mails: rieko.verhagen@philips.com (R.V.); A.Hussain@utwente.nl (A.H.); boudot.clemence@gmail.com (C.B.); qt35@cornell.edu (Q.T.); dingsiqi.1@gmail.com (S.D.); j.a.holz@amc.uva.nl (J.A.H.); natallia.uzunbajakava@philips.com (N.E.U.)

**Keywords:** radial polarization, birefringence, confocal microscopy, polarization dependent contrast

## Abstract

The polarization characteristics of birefringent tissues could be only partially obtained using linearly polarized light in polarization sensitive optical imaging. Here we analyze the change in polarization of backscattered light from birefringent structures versus the orientations of the incident polarizations using linearly, circularly and radially polarized light in a cross-polarized confocal microscope. A spatially variable retardation plate composed of eight sectors of λ/2 wave plates was used to transform linearly polarized light into a radially polarized light. Based on the experimental data obtained from *ex-vivo* measurements on human scalp hairs and *in-vivo* measurements on hair and skin, we exemplify that the underestimation of the birefringence content resulting from the orientation related effects associated with the use of linearly polarized light for imaging tissues containing wavy birefringent structures could be minimized by using radially polarized light.

## 1. Introduction

Polarized light confocal microscopy is a reliable and robust technique to provide non-invasive real-time optical imaging of biological tissue at high resolution and contrast. By using cross-polarized confocal detection (CPCLSM), the depolarized light back scattered from birefringent tissue structures can be imaged with better contrast and resolution [1,2]. However, when tissues containing wavy birefringent structures are viewed with linearly polarized light, the change in polarization of backscattered light will show periodic variations depending on the orientations of the incident polarizations. This could result in underestimation of birefringence content and thus deteriorate the quality of information that CPCLSM can potentially offer. The dependence of change in polarization of backscattered light from birefringent medium on different incident polarizations was reported by Wang *et al.*, based on Monte Carlo algorithms [3,4]. They showed periodic variations in the degree of polarization of backscattered light versus the orientations of the incident linear polarizations for a linearly birefringent turbid medium (birefringence, δ > 0.001) [3]. Circularly polarized light has been proposed as a sensitive and specific means of evaluating birefringence content independent of the orientation [5]. However, the polarization state is not preserved in the focus due to change in polarization resulting from multiple reflections at dichroic mirrors and high NA focusing.

The development of methods for measuring polarization properties of biological samples has so far concentrated predominantly on beams with uniform states of polarization, and space variant polarization states have received less attention. However, new effects and phenomena have been recently predicted and observed for spatially inhomogeneous state of polarizations. Beams with spatially distributed polarization states have attracted high interest in recent years due to their unique properties and symmetry, which can be utilized in a variety of applications [6–10]. The unique properties of radially and azimuthally polarized laser beams to produce strong longitudinal electric and magnetic fields at the focus under tight focusing conditions are exploited in a great variety of applications, ranging from microscopy [10] and material processing [11] to optical trapping [12] and has been recently proposed for use in particle acceleration experiments [13]. For example, by spatially varying the polarization, the field distribution in the focal region can be adjusted to decrease in the lateral spot size, which is important for high resolution applications, such as detection of lines in optical recording and for linear optical traps. The use of spatially inhomogeneous radially and azimuthally polarized light may offer advantages and additional possibilities for measuring polarization properties of biological samples in polarized light confocal microscopy.

Recently, we have shown that cross-polarized confocal laser scanning microscopy, when combined with variation of the polarization of the incoming light, can be used to enhance the contrast between the birefringent hair cortex and the non-birefringent skin [14,15]. In this manuscript, we for the first time report on the orientation related effects of linearly, circularly and radially polarized light on the change in polarization of backscattered light from birefringent structures using *ex vivo* measurements on human scalp hairs and *in vivo* measurements on hair and skin in a cross polarized confocal microscope.

## 2. Experimental Section

### 2.1. Generation of Radially Polarized Light

We have transformed a linearly polarized homogenous beam into radially polarized light using a Spatially Variable Retarder (SVR) [16,17]. The SVR is circular and consists of eight pieces of a half lambda wave-plate (multiple order antireflection coated for 830 nm) with each sector with different orientation of the “slow” axis of the birefringent crystal. The pieces of the SVR are made by dicing two half lambda wave-plates each into eight pieces [16]. When the linearly polarized beam passes through the SVR, each sector rotates the polarization vector to a different orientation angle. The polarization distribution after passing through the SVR is expected to be close to nearly radially polarized, except for the air gaps between each sector.

### 2.2. Spatially-Resolved Polarization Measurements

The experimental configuration used for spatially resolved measurement of Stokes parameters is based on the principles of circular polarimetry [18,19]. The polarimeter consists of a polarizer and a quarter wave plate with optical axes making an angle of 45° with respect to each other to make a circular polarizer. A CCD camera (Marlin F145B, Allied Vision technologies, Germany) is used to record the intensity of light after passing through the combination of the quarter wave-plate and the polarizer. Each pixel (6 × 6 μm^2^) on the CCD camera is considered as an individual detector and the camera can provide up to 30 frames per second with full resolution. The polarizer and the quarter wave-plate are fixed in a rotating mount for precise angular rotation of the components around their optical axis independent of each other. The rotation of the circular polarizer (*i.e.*, the combination of the quarter wave-plate and the polarizer) around its optical axis and the flipping of the circular polarizer by 180° are performed by two standard rotatory mounts (PR 50 compact high speed rotatory stage) controlled by stepper motors. In each step, 256 grey images are taken by the CCD camera and averaged to reduce internal noise in the CCD camera. The calculation of the Stoke parameters is done in Matlab. The experimental results are compared with the Stokes parameters calculated using Mueller matrix. An Eigen state generator consisting of a linear polarizer and a quarter wave plate was used to create known incident states of homogeneously distributed polarizations-linear polarization at −45° and circular polarization. Finally, the eigenstate generator was replaced by SVR for performing spatially resolved polarization measurements of radially polarized light.

### 2.3. Analysis of Linearly, Circularly and Radially Polarized Light

**Figure 1.**
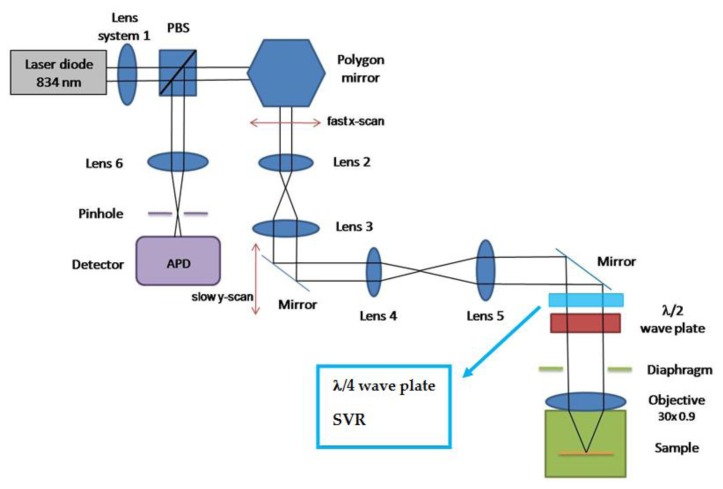
Schematic diagram of cross-polarized confocal laser scanning microscope used for *ex-vivo* and *in-vivo* measurements.

Hair and skin imaging experiments were carried out using a cross-polarized confocal laser scanning microscope (VivaScope 1000, Lucid Inc., Rochester, NY, USA) in reflection mode with a wavelength of 830 nm. The Schematic diagram of the experimental set-up is shown in Figure 1. The laser illumination beam was scanned by polygon and galvanometer mirrors and relayed into the water immersion microscope objective that focuses the beam into the sample. A water immersion microscope objective (30×, NA = 0.90) corrected for the cover glass was used to focus the laser light onto the sample. The maximum field of view was 450 μm × 400 μm. Light reflected from the tissue was collected by the objective, descanned, and focused onto a pinhole (50 μm). Light passed through the pinhole illuminated a silicon avalanche photodiode. The detected optical intensity was digitized by an 8-bit frame grabber and saved as a bitmap image using Labview.

### 2.4. Ex-Vivo *and* In-Vivo Measurements

The *ex vivo* experiments were performed on non-medullated Indian black scalp hair and *in-vivo* experiments were performed on black hair on skin type V. Cross-polarized confocal images were measured at different focusing depths inside hair and skin for different orientations of the incident polarization with respect to the optical axis of the hair. To compare quantitatively the dependence of contrast on the different orientations of the incident polarizations, the variation of contrast is calculated as a function of focusing depth as follows:

(1) $$\text{Variation of contrast}(R)=\frac{\left(\overset{{I_{B}}^{P1}}{\;}/\underset{{I_{N}}^{P1}}{\;}\right)}{\left(\overset{{I_{B}}^{P2}}{\;}/\underset{{I_{N}}^{P2}}{\;}\right)}$$

where I_B_ and I_N_ represents the intensity detected from the birefringent sample (hair) and non-birefringent background (air or skin). P1 and P2 correspond to two orientations of incident polarization with an orientation difference of 45°. The dependence of hair-skin contrast on hair orientation will be minimum for R = 1. The image analysis is performed in Matlab. In each image, three areas corresponding to 25 × 25 μm^2^ (35 × 30 pixels) were randomly selected for calculating the average intensity in the selected area. The mean and the standard deviation of the intensity detected were then calculated. The data were normalized with respect to the optical power used during the experiment.

## 3. Results and Discussion

### 3.1. Generation of Radially Polarized Light

**Figure 2.**
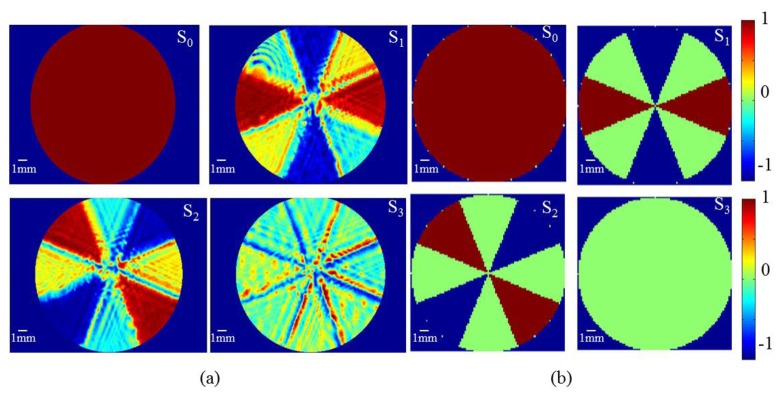
(**a**) Experimental and (**b**) Calculated stokes parameters at each point for incident radially polarized input beam.

Spatially resolved polarization measurements performed for homogeneous input beams show that the Stokes parameters at each location within the beam can be determined with accuracy higher than 98% at each transverse point in the observation plane. The spatial resolution is limited only by the pixel size (6 × 6 μm^2^) of the detection camera. The radial polarization distribution measured is represented in terms of Stokes parameters in Figure 2a and the results are compared with those calculated using Mueller's matrices plotted in Figure 2b. The results clearly indicate that the input beam is nearly radially polarized, except for the regions between each sector. The Stokes parameters measured for +45 and −45 degrees are not exactly same. This is probably due to the relative change in the orientation of different sectors with respect to each other and the inaccuracy while cutting segments from different wave plates. In addition, small fraction of the light passes through the air gaps in-between the sectors has different polarization than the light passing through the SVR. Light which passes through the gaps gets diffracted from the edges and contributes to the noise seen in Figure 2a.

### 3.2. Analysis of Linearly, Circularly and Radially Polarized Light in CPCLSM

**Figure 3.**
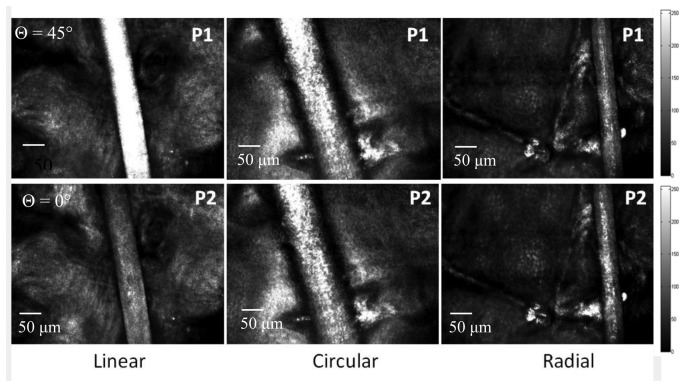
CP-CLSM images of non-medullated hair and skin (*in-vivo*) measured with linear, circular and radial polarizations for two orientations of the incident polarization (P1 and P2).

Cross polarized confocal images of non-medullated Indian black hairs measured using linearly, circularly and radially polarized light are shown in Figure 3 for different orientation of the incident polarization with respect to the optical axis of the hair. The effect of birefringence on the propagation of linearly polarized light is dependent on the angle between the incident polarization and optical axis.

**Figure 4.**
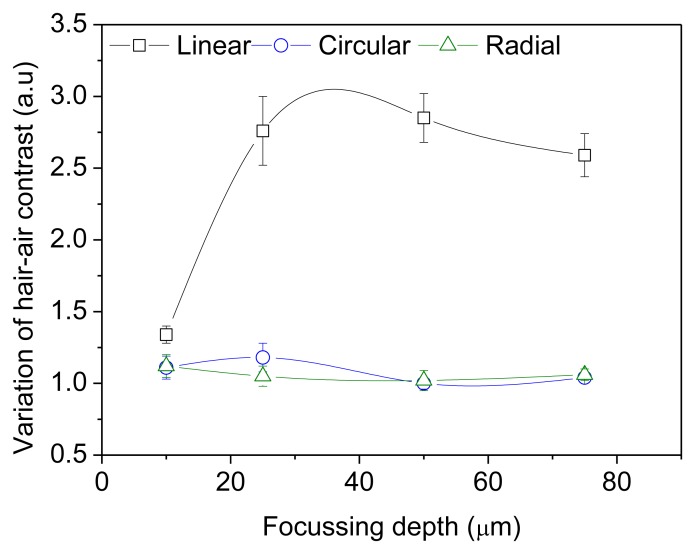
Variation of hair-air contrast measured on non-medullated black hair (*ex-vivo*) as a function of focusing depth inside hair for different orientations of incident polarizations.

**Figure 5.**
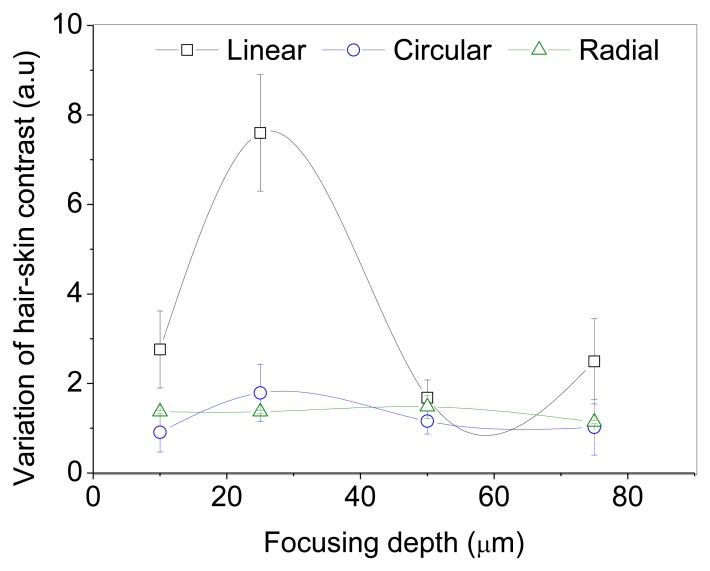
Variation of hair-air contrast measured on non-medullated black hair (*in-vivo*) as a function of focusing depth inside hair for different orientations of incident polarizations.

The ratio contrasts (R) measured for different orientations of the incident polarization with respect to the optical axis of the hair are shown in Figures 4 and 5. In *ex vivo* and *in vivo* measurements, the contrast variation is high for linear polarization. The intensity counts detected from the hair is significantly low when the hair axis is at 0° or 90° with respect to incident polarization. However, the effect of birefringence on the propagation of circularly and radially polarized light is not angle dependent (R = 1) since there is no preferential direction of orientation.

The hair consists of a thin outermost layer, the cuticle, a porous central part, the medulla, and the cortex. The cortex of the human hair is birefringent, where the ordinary and extraordinary refractive indices, n_0_ and n_e_, are 1.541 and 1.548, respectively [14]. The intensity of the light originating from hair strongly depends on cortex birefringence value (δ), optical path length (x) and the orientation of the incident polarization. The birefringence value, defined as the difference in refractive index between the extraordinary and ordinary rays travelling through the anisotropic cortex is equal to 0.007. The intensity reached the minimum and maximum when the orientation of the incident polarization is at 0° or 90° and at 45° with respect to the optical axis of the birefringent material (hair), respectively. If the angle between the hair axis and the incoming light polarization is 0° or 90°, the light traversed through the hair experiences no birefringence and consequently no change in polarization (apart from that induced by light reflectance at the hair interface, which is described by Fresnel equations). Hence, the amount of light originating from a hair detected in a cross-polarized mode will be minimal. But if the polarization of the incoming light is at 45° with respect to the hair axis, light traversing through a hair will experience birefringence, which results in a change in polarization. The maximal birefringence effect observed for polarization orientation at 45° with respect to the hair axis is in agreement with the known statement that one of the hair optical axes is along the hair itself.

The incident polarization state changes with propagation depth within a birefringent medium. The phase retardation, d, between orthogonal polarization components is proportional to the optical path length traveled through the birefringent medium. For the backscattered photons detected from larger focusing depths, depolarization can occur from multiple scattering. The degree of polarization of incident light tends to approach zero as the optical path length increases. The reduction in the variation of contrast with increasing depth is due to depolarization from multiple scattering and a decrease in the signal to noise ratio.

Here we demonstrate that the use of cross polarized confocal microscopy with radially polarized light has the ability to enhance the birefringent assessment of tissue, independent of its angular orientation. This is useful for clinical and diagnostic applications where it is not possible to orient the specimen of interest to the examiners preference. However, tightly focused radially polarized beam has pronounced side-lobes, resulting in lower energy density in the focus. This may lead to lower intensity in the confocal images while using radially polarized light compared to linearly and circularly polarized light [20].

### 4. Conclusions

In this manuscript, we report for the first time on the change in polarization of backscattered light from birefringent structures *versus* the orientations of the incident polarizations using radially polarized light in a cross-polarized confocal microscope. The results are compared with linearly and circularly polarized light. Spatially variable retarder composing of eight sectors of λ/2 wave plates was used to transform a linearly polarized homogenous beam into radially polarized light and we have measured a nearly-radial polarization distribution using this in-house polarimeter. The experimental data obtained using *ex-vivo* and *in-vivo* measurements on human scalp hairs and human skin in cross-polarized confocal microscope demonstrate that the underestimation of the birefringence content resulting from the orientation related effects associated with the use of linearly polarized light for imaging tissues containing wavy birefringent structures could be minimized by using radially polarized light.

We aim to further develop this method for depth resolved measurements of Stokes parameters in a polarization sensitive confocal microscope. This has potential applications in imaging simultaneously the structural properties and their effects on the polarization state of backscattered light. The compensation for the depolarization effects in the optical path using a spatial light modulator and later with a fixed phase plate structure is expected to increase the sensitivity of the method for imaging the birefringence in the biological samples.
